# Survival from non-Hodgkin lymphoma in England and Wales up to 2001

**DOI:** 10.1038/sj.bjc.6604606

**Published:** 2008-09-23

**Authors:** P W M Johnson

**Affiliations:** 1 NCRI Lymphoma Clinical Studies Group, CR UK Clinical Centre, Somers Cancer Research Building, Southampton General Hospital, Southampton SO16 6YD, UK

Non-Hodgkin lymphoma includes a heterogeneous group of illnesses. The presentation may range from an isolated and non-enlarging lymph node asymptomatic for many years, to massive compromise of vital organs by infiltration that proceeds with great speed. Further complexity is added by the variety of anatomical sites which may be affected: most NHL involve nodal and bone marrow disease, but significant numbers may involve the brain, meninges, lungs, gastrointestinal tract, liver, kidneys, skeleton, skin or other sites. In each case, there are distinctions of prognosis according to the histologic grade and type.

During the period studied, there has been a well-documented rise in the incidence of large B-cell lymphoma in patients more than the age of 65 years (Hartge *et al*, 2004). This, together with the ageing population generally has led to a marked rise in the number of older patients with aggressive lymphoma for which only relatively intensive chemotherapy will be curative. There has also been an increase in the number of lymphomas associated with impaired immunity, whether because of HIV or immunosuppression following organ transplantation. In addition, there are recognised geographical differences in the incidence of different lymphoma types; hence the picture is susceptible to the effects of immigration as well.

The diagnosis of lymphoma depends upon a tissue biopsy and its histology. The clinical presentation of the illnesses has not changed greatly over time, with most appearing as nodal masses, and some with manifestations of extra-nodal disease. There have been notable advances in the techniques used to subdivide lymphoma over the last 20 years, in particular monoclonal antibodies to determine the cell of origin, whether B- or T-lymphocyte, and the expression of a variety of cellular markers to distinguish all the various categories within these, related to some extent to their non-malignant counterparts in lymphocyte ontogeny (Harris *et al*, 1999). Further refinement has come from the use of cytogenetics to identify characteristic chromosomal rearrangements, and molecular techniques to define the precise nature of them, for example, the *t*(14;18)(q32;q21) rearrangement of the *Bcl-2* gene which is most often seen in follicular lymphoma, or the *t*(11;14)(q13;q32) rearrangement of Cyclin-D1 seen in mantle cell lymphoma. Yet, further refinement of the process is now being achieved with the use of gene expression profiling to build up detailed maps of genetic signatures, which have prognostic, and potentially therapeutic significance (Alizadeh *et al*, 2000; Rosenwald *et al*, 2002).

All these techniques have given a level of sophistication to lymphoma classification, which has been helpful for planning treatment and trials, but has had the less welcome effect of making previous classifications redundant, and to some extent irrelevant. The result is that beyond broad categories of ‘aggressive’ and ‘indolent’ it is difficult to make direct comparisons of the case mix over the period under study. Given the very different prognoses for the various subtypes of lymphoma this renders longditudinal data of limited use.

## Treatment

The variety of lymphoma types is mirrored in a range of approaches to treatment. Thus, localised low-grade disease, such as follicular, marginal zone or lymphocytic lymphoma may be managed by excision alone, with or without local radiotherapy, and disseminated low-grade disease is frequently managed expectantly, until there is a specific indication for treatment, such as a fall in the blood count, progressive nodal enlargement, systemic symptoms or compression of internal organs. Conversely, high-grade disease, such as diffuse large B-cell or Burkitt lymphoma is usually treated with intensive combination chemotherapy, sometimes using consolidation radiotherapy to the sites of residual disease. Myeloablative treatment may be used for recurrent disease, with autologous progenitor cell rescue of the marrow, and in some subtypes allogeneic transplants are used.

There have been significant developments in lymphoma therapy during the period studied, although the rate of change has probably increased more since 2000, making it likely that the rise in survival figures will continue and perhaps accelerate.

The biggest single change in lymphoma therapy in the last decade has been the introduction of treatment with monoclonal antibodies, in particular those recognising CD20 on the B-cell surface. Clinical trials conducted since the late 1990s have shown marked benefit from the incorporation of anti-CD20 into treatment, both as a single agent and in combination with chemotherapy. Only a small minority of patients received such antibodies before 2000; hence it is unlikely that this development influenced the figures greatly. There is now strong evidence of a survival benefit from the use of combination chemoimmunotherapy in the initial treatment of follicular (Herold *et al*, 2003; Forstpointner *et al*, 2004; Marcus *et al*, 2005) and diffuse large B-cell lymphoma (Coiffier *et al*, 2002; Habermann *et al*, 2006), whereas lesser effects are seen in mantle cell lymphoma, amounting to prolongation of remission without substantial impact on overall survival (Lenz *et al*, 2005). Trials are ongoing to determine the effects in the less common B-cell lymphomas, such as Burkitt's, lymphoplasmacytic and marginal zone types. Further developments, such as the use of anti-CD20 conjugated to radionuclides for the selective delivery of radiation are still being tested, as is the use of anti-CD20 maintenance therapy, which seems to convey benefit in follicular lymphoma, at least in second remission (van Oers *et al*, 2006).

The other major development in lymphoma treatment during the period of study was the introduction of high-dose therapy, with the aim of overcoming chemoresistance by administration of myeloablative doses, followed by rescue using previously collected haemopoietic progenitor cells. At the beginning of this period, the progenitor cells were collected in bone marrow aspirates, but from the early 1990s there was a progressive switch to the use of peripheral blood progenitors, mobilised by chemotherapy followed by the use of granulocyte colony-stimulating factor and harvested by leukapheresis. This approach has the advantage of shortening the time to re-engraftment and recovery, with consequent improvements in treatment-related mortality, and the capacity to carry out such procedures in older or less fit patients. There is evidence of survival benefit from high-dose therapy in the second remission from both diffuse large B-cell lymphoma (Philip *et al*, 1995) and follicular lymphoma (Brice *et al*, 2000; Schouten *et al*, 2003), whereas in mantle cell lymphoma the data suggest prolongation of remission without an effect on survival (Dreyling *et al*, 2005). High-dose therapy has also been used, with some suggestion of benefit, as part of initial therapy for lymphoblastic (Sweetenham *et al*, 2001) and peripheral T-cell lymphomas (Rodriguez *et al*, 2003), both types with a relatively poor outlook after conventional treatment. Allogeneic transplants, which depend upon engraftment of marrow from an HLA-matched donor have been less commonly used in lymphoma. They have more complex toxic effects, particularly graft-versus-host disease, and the treatment-related mortality can be up to 30%, but they may offer cures to the small number of patients fit enough to have them carried out for low-grade lymphoma, which would otherwise pursue a course of serial recurrences (Morris *et al*, 2004). There is, however, little evidence from prospective clinical trials as yet.

The organisation of clinical services for lymphoma has largely followed a dispersed model in England and Wales, with most district hospitals managing cases locally, only referring to specialist centres for uncommon types or those requiring intensive and high-dose therapy. The National Institute for Clinical Excellence published guidelines for the organisation of services in haematologic malignancy in 2003; hence, these cannot have directly influenced the data presented here, although there were already changes taking place in service delivery before publication of the guidelines, particularly central pathology review. The need for more complex diagnostic subclassification of NHL has increased as the role of treatments specific to particular entities has emerged, for example, diffuse large B-cell lymphoma must be rapidly distinguished from Burkitt lymphoma to achieve the optimum outcome in both types. In clinical trials, the results of treatment seem to differ little between smaller units and specialist centres, although the proportion of patients entering such trials varies greatly, and nowhere is above 10% of incident cases.

## Discussion

The modest rise in survival figures over the period studied is encouraging, although as already mentioned, it seems likely that the rate of improvement may increase after 2000. The progress made may be underrepresented, as the case-mix has changed through the rising incidence of aggressive lymphoma in the older population. The outcomes in this group are uniformly worse than in younger patients, principally because the delivery of effective treatment is often compromised by comorbidity. The rising incidence of immunodeficiency-related lymphomas, which again are more difficult to treat effectively, will also have had a small negative effect.

The increments in survival during this period probably reflect the more effective application of existing chemotherapy treatments and better supportive care, particularly with antibiotics, blood products and growth factors to manage the consequences of myelosuppression during chemotherapy. Myeloablative therapy for recurrent disease, although conveying survival benefits in clinical trials is applicable only to a small minority of patients, probably no more than 5% of the total with NHL; therefore is unlikely to have had a major impact on survival across the whole population.

The differences in outcome among the deprivation groups are in keeping with the trends seen in most other tumour types. It is likely that comorbidity, particularly related to cigarette smoking, plays a significant role in this difference, as the outcome in NHL is more dependant upon ability to withstand intensive systemic treatment than for many tumour types. Patients with aggressive lymphoma with significant cardiorespiratory illnesses are much more prone to death as a result of treatment toxicity, or recurrent lymphoma following premature cessation of therapy owing to life-threatening complications or cumulative side effects. It is also likely that the competing risks of cardiorespiratory or other illnesses, more prevalent in deprived groups, are heightened by the effects of systemic treatment in the relatively elderly lymphoma population. Nutritional state is another determinant of the capacity to receive intensive chemotherapy, and this too will act to disadvantage the more deprived groups. Unfortunately there is little clear evidence on these factors, as clinical trials usually exclude patients unfit for intensive therapy. Other factors, such as late presentation and reduced access to specialist services probably play a less dominant role in the differences according to deprivation: in general the dispersed model of lymphoma services has produced good population-wide access to care and there is little evidence to link time to diagnosis with outcome in NHL. Aggressive lymphomas generally present with sufficiently severe symptoms to promote rapid referral, although survival with indolent lymphomas is little affected by the speed of therapy. An exception to this may be immunodeficiency-related lymphoma among immigrant populations. Outcomes in this group are likely to be worse both because the risks of treatment are greater and because of reduced access to complex medical interventions.

In conclusion, the rising survival figures are welcome, and likely to underrepresent the progress being made owing to changes in case-mix. There are good reasons to expect the improvement to continue and accelerate as monoclonal antibodies and new small molecule therapies enter wider use. The differences in outcome between wealthier and more deprived groups remain a source of concern, but there are reasons to expect that these may begin to decline as supportive care improves and cigarette smoking becomes less prevalent. The implementation of NICE guidelines for care of haematologic malignancy will largely consolidate a process, which was already underway previously, but there may be further benefits in terms of better histopathologic characterisation that becomes more important as the specificity of treatment increases.
